# Impulsivity and emotion regulation in medical students

**DOI:** 10.1192/j.eurpsy.2023.1552

**Published:** 2023-07-19

**Authors:** N. Halouani, M. Daoued, O. Elleuch, M. Turki, S. Ellouze, J. Aloulou

**Affiliations:** Psychiatry B, Hedi Chaker University Hospital of Sfax, Sfax, Tunisia

## Abstract

**Introduction:**

Impulsivity in adolescents has been largely studied as it is frequently observed in that phase. However, the relationship between impulsivity and emotion regulation has been rarely explored.

**Objectives:**

Our study aimed to assess impulsivity and emotional regulation in medical students as well as to explore the link between them.

**Methods:**

This was a descriptive and analytical cross-sectional study conducted from September to December 2017, among first and second year students of the medical school of Sfax who were aged between 18 and 19 years. We collected sociodemographic as well as clinical data of the participants. “Barratt Impulsivity Scale” (BIS) and “Difficulties in Emotion Regulation Scale” (DERS) were used to assess impulsivity and emotion regulation respectively.

**Results:**

One hundred students were included in our study, with a mean age of 18 years and a sex ratio of 0.81. Among them, 62% were smokers with an average consumption of 19.6 packets year. Alcohol and cannabis use was reported by 9% and 5% of the students respectively.

The mean impulsivity score on the Barratt scale was 66.78 ± 9.44 with scores ranging from 40 to 112. Among our participants, 25% had a high level of impulsivity (score > 72). Unplanned impulsivity was the dimension with the highest mean score (23.74±4.64). Our results showed that impulsivity was significantly associated with the male gender (p=0.002) and smoking (p<10-3).

As for emotion regulation, the mean score on the DERS scale was 78.8 ±17. The majority of the students (64%) had a moderate difficulty in regulating emotions.

Our results showed a positive correlation between impulsivity and emotional regulation with a moderate link (p=10-3; r= 0.57).

The high emotion dysregulation group had a significantly higher score on the two dimensions of impulsivity: attentional impulsivity (p=10-3) and unplanned impulsivity (p=0.047).

**Conclusions:**

Our study highlights the relationship between emotion dysregulation and impulsivity, suggesting that emotion regulation may be an important factor to consider when assessing impulsive adolescents.

**Disclosure of Interest:**

None Declared

